# Improving Children’s Lifestyle and Quality of Life through Synchronous Online Education: The Nutritional Adventures School-Based Program

**DOI:** 10.3390/nu15245124

**Published:** 2023-12-16

**Authors:** Dimitrios V. Diamantis, Almog Shalit, Konstantinos Katsas, Evangelia Zioga, Dina Zota, Christina Maria Kastorini, Afroditi Veloudaki, Matina Kouvari, Athena Linos

**Affiliations:** 1 PROLEPSIS Civil Law Non-Profit Organization of Preventive Environmental and Occupational Medicine, 15121 Athens, Greece; d.diamantis@prolepsis.gr (D.V.D.); almogshalit@gmail.com (A.S.); k.katsas@prolepsis.gr (K.K.); l.zioga@prolepsis.gr (E.Z.); d.zota@prolepsis.gr (D.Z.); a.veloudaki@prolespsi.gr (A.V.); a.linos@prolepsis.gr (A.L.); 2 Endocrine Unit and Diabetes Center, Department of Clinical Therapeutics, Alexandra Hospital, School of Medicine, National and Kapodistrian University of Athens, 11528 Athens, Greece; 3 Medical School, National and Kapodistrian University of Athens, 11527 Athens, Greece; 4 Nutrition Department, ‘Sotiria’ Chest Diseases Hospital, 11527 Athens, Greece; ckastorini@yahoo.gr; 5 Department of Nutrition and Dietetics, School of Health Science and Education, Harokopio University, 17676 Athens, Greece; 6 Discipline of Nutrition and Dietetics, Faculty of Health, University of Canberra, Canberra, ACT 2601, Australia; 7 Functional Foods and Nutrition Research (FFNR) Laboratory, University of Canberra, Bruce, ACT 2617, Australia

**Keywords:** nutritional education, school-based programs, Mediterranean diet, children, quality of life

## Abstract

The early introduction of effective nutritional educational programs is pivotal for instilling sustainable healthy behaviors. The present work aims to present a best practice example of a nutrition and overall lifestyle school-based training program, the Nutritional Adventures (“Diatrofoperipeteies”). Conducted during 2020–2022 in Greek primary schools, this synchronous, online educational initiative included two 1-school-hour activities with a nutrition instructor. Additionally, schools were randomly assigned to supplementary “at-home” supported-by-parents or “in-class” supported-by-educators educational activities. In total, *n* = 12,451 students of 84 primary schools participated. Parent-completed questionnaires were selected in the recruitment and post-intervention phase (40% participation rate); overall, the working sample was *n* = 1487 students. In the post-intervention phase, a significant increase in Mediterranean diet adherence was observed (KIDMED score: mean increment = 0.25 units; *p* < 0.001), particularly fruit and vegetable consumption. Time spent on physical activity increased, while screen time decreased. Students’ total quality of life significantly improved (PedsQL; mean increment = 1.35 units; *p* < 0.001), including on all of its subscales (physical, emotional, social, and school function). Supplementary educational activities that were supported by educators rather than parents yielded a more favorable impact on students’ lifestyle and quality of life. The Nutritional Adventures program can be regarded as a successful initiative in primary schools, yielding immediate advantages that extend beyond promoting healthy dietary habits.

## 1. Introduction

A considerable transition from traditional healthy eating habits, such as the Mediterranean diet, to Western diets worldwide characterizes modern-day living. In Greece, this shift is evident, particularly with younger children, with approximately half of the students being poor adherers to the Mediterranean diet, while only one in twenty children has optimal adherence [1]. The same trend has been reported across all age groups in Mediterranean countries [2,3]. Owed broadly to globalization and industrialization, this shift towards energy-dense, nutrient-poor foods that are high in saturated fats, sugars, and salt [4] has profound consequences on global and individual health, considering that suboptimal diet has been ascribed with more deaths than any other risk factor globally [5].

The Mediterranean diet is a dietary pattern that is renowned for its benefits in lowering the risk of chronic disease and overall mortality, as well as at reducing obesity rates in both adults and children [6]. Such outcomes are achieved through a high intake of plant-based foods, including a high intake of fiber and low-glycemic-index carbohydrates and antioxidants through daily consumption of whole-grain unprocessed products, legumes, fruits, and vegetables, along with monosaturated and Omega-3 polysaturated fatty acids, mostly attributed to a high intake of olive oil and nuts and fish, respectively [7,8]. Additionally, red meat consumption is limited, while alcohol and particularly wine can be consumed in moderation [7,8].

Alarmingly, there have been consistent increases in the prevalence of overweight and obesity in early life stages, with nearly one in three school-aged children living with impaired weight status. On top of this, very recently, the World Health Organization (WHO) European Childhood Obesity Surveillance Initiative underscored that not a single Member State of the Region is on track to reach the target of halting the rise in obesity by 2025 [9]. To address this issue, more effective public health initiatives that promote healthy dietary and overall lifestyle habits are demanded. To address this issue, school-based nutrition education programs effectively promote adequate growth and improve childhood physical, social, and mental health while simultaneously setting the foundation for healthy habits throughout adult life [10]. According to the guide by WHO, educational programs should be child-centered, promoting active participation, with a planned curriculum that is appropriately designed for the different developmental stages [10]. Successful in-person school initiatives, with some incorporating digital components, have been implemented in multiple countries [11,12,13,14,15,16,17,18,19,20,21]. However, there is a limited presence of research on initiatives that are primarily based on delivering nutrition education sessions through digital platforms. Moreover, the inherent difficulties around implementation in remote and isolated communities highlight the need for a more universally accessible solution. The COVID-19 pandemic familiarized the educational system and students with online platforms, presenting new possibilities.

The “Nutritional Adventures” program (“Diatrifoperipeteies”) is a synchronous, online educational program generated during the COVID-19 pandemic to promote healthy nutrition and lifestyle in students in primary schools across Greece, with no geographic restrictions. The primary objective of the present work was to evaluate the effectiveness of the “Nutritional Adventures” program in encouraging students to adapt to healthier dietary habits. Secondary research hypotheses were also investigated, in particular (a) if the suggested program may contribute to improving students’ overall health-related quality of life (HRQoL) and (b) if the program’s effectiveness is affected by the different training methods and means used.

## 2. Materials and Methods

### 2.1. Study Sample and Setting

The “Nutritional Adventures” program was implemented during the school years 2020–2021 and 2021–2022 at primary schools in Greece. A random selection of schools was performed using the national list that the Greek Ministry of Education provided. Only primary schools were considered eligible for this program, since its educational content was tailor-made for this grade. Due to the online character of the educational activities, no geographical constraints were set. In-class Internet access and laptop or desktop computers were the minimum technological prerequisites needed. Once a school was randomly selected, an information letter with the program scope, content, and specific activities was delivered. The administration body of the school, led by the principal, was responsible for accepting or rejecting the invitation to participate in the program. In case of a lack of approval, another school was randomly selected, and the same process was followed. During the 2020–2021 and 2021–2022 school years, the program reached *n* = 84 schools and *n* = 12,451 students across Greece. 

### 2.2. Bioethics

The “Nutritional Adventures” program was approved by the Ethical Committee of Prolepsis Institute (13913-n.3, on October 2021) and is being conducted in accordance with the Declaration of Helsinki. The program was implemented in schools after the official approval of the school administrative body as well as the signed consent forms of students’ parents. Parents answered the questionnaires in pre- and post-intervention phases. All questionnaires were anonymous with no potential for deidentification. Accurate matching of pre- and post-intervention responses was accomplished by a set of sociodemographic characteristics.

### 2.3. The “Nutritional Adventures” Program

The “Nutritional Adventures” program is a synchronous, online educational intervention, tailor-made for students in primary schools and implemented by instructors with expertise in the field of nutrition and public health promotion in early life stages. 

#### 2.3.1. The Program Objective

The Nutritional Adventures program is a 1-month school-based educational initiative that aims to promote healthy living and dietary habits in primary school children. 

#### 2.3.2. The Program Framework

The “Nutritional Adventures” program consists of two sections. The first section is related to a synchronous, online educational program implemented at classes of primary schools by an instructor who is specialized in the field of nutrition and public health promotion in childhood. Two 1-school-hour online sessions are delivered in the form of interactive lectures. The window time between the 1st and 2nd session is two to three weeks. During this timeframe, students are assigned specific tasks related to the training that they received.

#### 2.3.3. The Program Experimental Design

##### Training Content

An interdisciplinary team of nutritionists/dieticians, psychologists, health promotion specialists, and pedagogues was responsible for the training content of the program. The training content was externally approved by the National Institute of Educational Policy and implemented under the auspices of the Greek Ministry of Education and Religious Affairs. The Syllabus of the program includes primarily topics related to the dietary guidelines in childhood, the different food groups and their nutritional value, and the meals throughout the day. Key messages on thematic areas related to physical activity, food safety, and food waste are also included. The National Dietary Guidelines for infants, children, and adolescents in Greece was used as the main scientific resource [22]. 

##### Adaptation of the Program to Educational Grade

The training content, means, and methods were adapted to the educational grade. In particular, two versions of the program were designed and tailor-made for 1st–3rd Grade and 4th–6th Grade. In younger students (i.e., 1st–3rd Grade) the story-telling educational approach was used based on a nutrition fairytale named “The Nutritional Adventures in the Land of Delicious Kingdoms”, which was designed in the context of the DIATROFI food aid and healthy nutrition promotion Program and approved by the National Institute of Educational Policy as well as the Ministry of Education and Religious Affairs. The students were asked to investigate “Delicious Kingdoms”—each one corresponding to one food group—and resolve the puzzle of healthy nutrition. In older students (i.e., 4th–6th Grade), interactive training sessions were delivered to the students. Practical assignments were provided in 60% of the training, including crossword puzzles, exercises that combined knowledge quizzes and movements, recall of favorite meals, etc. 

##### Supportive Educational Material and Randomization of Training Methods

In all cases, students were provided with supportive educational material—tailor-made for their age—in the form of booklets and diaries. Students had the option to use it for “in-class” or “at-home” activities supervised by educators or parents, respectively. To examine the existence of superiority or equivalence in these two training approaches in relation to their effect on students’ dietary habits and overall HRQoL, a block randomization of the schools was performed (block size = 10).

### 2.4. Pre- and Post-Intervention Assessment

A structured questionnaire was used to record the sociodemographic characteristics of students and their families, their dietary and other lifestyle habits, and overall HRQoL. The questionnaires were completed by a parent or another guardian if a parent was not available. To assess the efficacy of the program, questionnaires were delivered once the school was recruited for the program (baseline), as well as after the completion of the program (follow-up). The follow-up questionnaire recorded parents’ perceived impact, acceptability, and satisfaction with the program. In total, *n* = 5043 baseline questionnaires were selected (40% response rate). The working sample of the present work was *n* = 1487 unique students, corresponding to *n* = 1487 paired pre- and post-intervention questionnaires; *n* = 3556 baseline questionnaires were excluded from the analysis due to the lack of completed follow-up questionnaires or due to inadequate demographic data to achieve accurate matching with the selected follow-up questionnaires. 

#### 2.4.1. Sociodemographic Characteristics

Sociodemographic characteristics included students’ age, sex, family structure, parents’ country of origin, level of education, and employment status. The level of education was classified as low (<9 years of education), moderate (9–12 years of education), and high (>12 years of education).

#### 2.4.2. Dietary Habits, Physical Activity, and Screen Time

Students’ dietary habits and overall level of adherence to Mediterranean diet were assessed using the KIDMED score [23]. This score consists of 16 questions that examine habits that are consistent with the Mediterranean dietary pattern. Each question is scored with either +1 or −1, depending on its connotation (e.g., “has cereal or grains (bread, etc.) for breakfast”, “skips breakfast”, respectively). The level of adherence is estimated from the total score and defined as “low” for a score ≤ 3, moderate for a score between 4–7, and “good” for a score ≥ 8. Physical activity and screen time were assessed using questions regarding the weekly hours devoted to physical activity and the weekly hours of extracurricular screen time.

#### 2.4.3. Health-Related Quality of Life

In order to assess the student’s HRQoL, the PedsQL 4.0 measurement model was utilized [24]. The tool has been validated for the Greek population and evaluates four generic core scales (physical functioning, emotional functioning, social functioning, school functioning) and summary scores, with a total of 23 questions. Each question is scored on a scale of 0 to 4, with answers ranging from “never a problem” (score 0) to “always a problem” (score 4). All scores are reversed and linearly transformed to a scale of 0–100, in which high scores translate to better self-perceived conditions. The total HRQoL is calculated as the mean of all core scales and scored on a scale of 0–100.

#### 2.4.4. Body Mass Index

Parents were asked to report their children’s weight and height. The revised International Obesity Task Force cut-offs according to the pooled Lambda Mu and Sigma curves were used for Body Mass Index (BMI) classification (underweight, normal, overweight, obese) [25]. Underweight, overweight, and obese students were classified as students with “unhealthy weight”.

### 2.5. Statistical Analysis

Categorical variables are presented as relative frequencies (%), and continuous variables are presented as mean values (standard deviation). Student’s *t*-test for independent samples was used to compare continuous variables among the different groups. The normality of the continuous characteristics’ distribution was tested through the P-P plot and the Shapiro–Wilk test. To compare dichotomous variables, a chi-square test was performed. To compare differences between participants’ baseline and follow-up characteristics, the paired Student’s *t*-test was used. For the data analysis, the statistical package for social sciences (IBM SPSS, Chicago, IL, USA) version 20.0 was used, and a *p*-value of ≤0.05 was regarded as statistically significant.

## 3. Results

### 3.1. Paternal and Student Demographic Characteristics

Students’ sociodemographic characteristics according to their grades are presented in Table 1. About half (54.4%) were 1st–3rd grade students. The mean age was 9 ± 2 years, 45.1% were male, and 25.3% had two or more siblings. Most parents were from Greece (84.4% of fathers; 82.8% of mothers), while approximately half of the fathers and 70% of the mothers were assigned to the high educational group. The demographic characteristics did not differ significantly between 1st–3rd grade and 4th–6th grade students, apart from the expected differences in age and number of siblings.

### 3.2. Improvement in Dietary Habits and HRQoL According to Students’ Grade

The statistical analysis of the baseline and follow-up revealed a significant improvement in the KIDMED score (mean increment = 0.25 units; *p* < 0.001), as presented in Table 2. The significant increment remained, even when the adherence to the Mediterranean diet was classified into three groups, along with an improvement in daily fruit, vegetable, dairy, and sweet consumption (*p* < 0.05). Regarding their lifestyle behaviors, students increased their physical activity by 20 min on average weekly and reduced the screen time spent in front of television or video gaming by approximately 30 min (*p* < 0.001). Similar results were observed regarding KIDMED score, physical activity, and screen time between 1st–3rd grade students and 4th–6th grade students. The 4th–6th grade students reported a significant improvement in dairy consumption, whereas this improvement did not differ between baseline and follow-up in 1st–3rd grade students.

An elevated HRQoL (mean increment = 1.35 units), as well as better scores in physical (mean increment = 0.99 units), emotional (mean increment = 2.15 units), social (mean increment = 0.84 units), and school functions (mean increment = 1.58 units) was observed between the baseline and follow-up, as presented in Table 3 (*p* < 0.05). Similar results, except for physical function, were observed between 1st–3rd grade students and 4th–6th grade students. The 1st–3rd grade students reported a significant improvement of 1.56 units in their physical function, whereas the physical function of 4th–6th grade students did not differ between baseline and follow-up.

### 3.3. Improvement in Dietary Habits and HRQoL According to Intervention Group

Comparisons were also made between the parent and educator groups, as presented in Table 4 and Table 5. Students’ demographic characteristics were similar between the two groups, except for parental immigration status (12.7% of students in the parent and 18.2% of students in the educator group had an immigrant father; *p* < 0.05) (Table A1). Similar to Table 2, both intervention groups demonstrated a significant improvement in KIDMED (0.3 mean increment in parent group; *p* < 0.001, 0.2 mean increment in educator group; *p* = 0.002). However, a notable increment in daily fruit and vegetable consumption between baseline and follow-up was observed only in the parent group (*p* < 0.05). As for their lifestyle, significantly higher levels of physical activity and fewer hours of screen time during follow-up were revealed in both groups (*p* < 0.001).

As described in Table 5, both intervention groups demonstrated a significant increment in HRQoL score (0.98 mean increment in parent group; *p* < 0.001, 1.69 mean increment in educator group; *p* < 0.001), with similar results observed for emotional and school functioning. Physical and social function were improved significantly only in students classified as being in the educator group (p’s < 0.05). Notably, in each intervention group, the outcome was not affected by the grade grouping (Table A4), besides the physical functioning score in the educator group, which was more pronounced in the lower-grade students (mean increment = 2.25 for 1st–3rd grade students vs. mean increment = 0.14 for 4th–6th grade students; *p* = 0.008).

Intervention groups were also compared for all outcomes separately for each grade group. Parent–educator demographics were similar in both grade groups for most characteristics, except for students’ age and sex distribution and parental immigration status (Table A2). The educator group intervention proved more effective in the lower grades (1st–3rd) regarding total HRQL (parent group: mean increment = 1.07 units vs. educator group: mean increment = 2.08 units; *p* = 0.047) and physical functioning (parent group: mean increment = 0.81 units vs. educator group: mean increment = 2.25 units; *p* = 0.022). Physical activity was improved to a larger degree in the educator group (20.1%) compared to the parent group (11.3%) in the 4th–6th grade group (*p* = 0.003).

### 3.4. Improvement in Dietary Habits and HRQoL according to BMI Classification

Comparisons were also made between students with healthy and unhealthy weights, as presented in Table 6. Only students with an unhealthy weight demonstrated a significant improvement in KIDMED (0.3 mean increment; *p* = 0.005), as well as regarding the increment in daily fruit, vegetable, and fish consumption between baseline and follow-up (*p* < 0.05 for vegetable and fish; *p* = 0.07 for fruit). As for their lifestyle, significantly higher levels of physical activity and fewer hours of screen time during follow-up were revealed in both groups (*p* < 0.05).

As described in Table 7, both students with healthy and unhealthy weights demonstrated a significant increment in HRQoL score (0.71 mean increment in students with a healthy weight; *p* = 0.008, 1.65 mean increment in students with an unhealthy weight; *p* < 0.001), as well as for emotional and school function. In general, students with an unhealthy weight reported a significantly higher improvement in HRQoL score and its subscales (p’s < 0.05), except for school function. Social function did not change significantly in both groups, whereas physical function was improved only in students with an unhealthy weight. 

### 3.5. Acceptability, Satisfaction, and Perceived Impact of the Nutritional Adventures Program

When parents were asked to report on their satisfaction with the program, about 92.1% reported that the provided educational material was good/very good, 88.5% were satisfied with the educational activities, and 85.4% considered the quality of the educational activities to be good/very good. Moreover, 90.2% of parents considered that their child perceived the program to be of high quality, and 96.2% advocated for the program to be implemented again next year in the school.

As for the perceived impact that the program had on their children, the vast majority quoted that their children improved their knowledge on nutrition (98.5%), improved their eating habits at school (88.3%), and requested healthier foods at home (82.4%). Parents also reported an improvement in their child’s eating habits (89.1%), and in particular, an increase in fruit (83.1%) and vegetable (79.4%) consumption, along with a limitation in unhealthy snack consumption (80.9%). 

## 4. Discussion

The “Nutritional Adventures” program, presented herein, implemented during the crucial period after the COVID-19 pandemic appeared to be a successful educational approach in promoting short-term healthy nutrition during early life stages with potential long-term effects. The present work—despite the effect of low magnitude—revealed a trend of improvement in a couple of dietary habits, as well as physical activity status. These observations—accompanied by other features of the program, including the participation of all students in the class and the collaboration in common nutrition projects with individual or group tasks, which enhanced students’ creativity as well as their interaction with educators and parents—had a positive impact on children’s HRQoL, especially school and emotional functioning. This implies the effectiveness of such initiatives beyond the scope for which it was initially designed. 

Online nutritional education for children is a valuable tool, fostering informed dietary choices and promoting healthy eating habits from an early age [26]. Through empowering critical thinking, well-informed children can make healthier food choices and improve their nutrition literacy [26,27], reducing the risk of non-communicable diseases, like obesity [28], and encouraging lifelong habits supporting physical and mental health [29]. Additionally, online education can cultivate children’s digital skills, developing proficiency in using digital resources, a crucial skill in the modern world [30,31]. Despite the prospect of web-based learning, its application in nutrition education for middle school children is limited, and primarily functions as a supplementary tool [32]. To our knowledge, the “Nutritional Adventures” program is the first exclusively virtual intervention in this demographic. The use of technology-centered interventions is more commonly integrated in adolescents, and has been successful at improving nutrition knowledge and a range of eating habits in the majority of studies [33]. The advent of online education has assumed heightened significance in the post-COVID-19 era, underscoring the importance of accessible, convenient/flexible, and expert-provided education and laying down the foundations for a more inclusive and effective online learning experience [27]. The resulting widespread availability of remote technology and increased digital literacy has facilitated the provision of educational services at the discretion of learners, without geographical constraints, reaching even remote areas at a reduced cost, thereby mitigating disparities in educational opportunities.

Online education can address the challenges related to the dissemination of credible information and provide expert-designed materials, especially in the context of the copious barriers in providing teacher-delivered nutrition education in schools [34,35]. Most elementary school teachers lack access to training resources and have limited training and expertise in healthy nutrition, along with limited motivation and capacity to provide nutrition education during school hours [34,35]. Moreover, the financial barrier to providing professional educational training for teachers may not align with the school’s professional learning goals [36]. Consequently, through online nutritional education, students, parents, and teachers can access high-quality, evidence-based information provided by expert nutritionists at a lower cost, allowing for more efficient resource allocation [27,37]. 

Opinions on the effectiveness of online education, compared to face-to-face, vary from ineffective to better than regular [38,39]. It is imperative to note that online learning can take many forms, each resulting in different outcomes [39]. Our program closely aligns with computer-assisted instruction, which integrates digital learning within the school framework under teacher and expert supervision, a form characterized by its beneficial outcomes, particularly in primary schools [39]. However, all online educational activities face multiple barriers, as face-to-face interaction is limited, and motivation from students to participate and teachers to deliver cannot be ensured [27,40]. Students and teachers that are less prone to use new remote technological means or with limited access to such means may face multiple technological difficulties and struggle with understanding and retaining knowledge [27]. Program designers are tasked with devising online education that can overcome these barriers by incorporating various strategies and characteristics into their training to ensure its effectiveness. 

Effective online education programs exhibit several key characteristics that render them proficient in instigating meaningful behavioral changes [27]. The WHO calls for a child-centered nutrition curriculum incorporating age-appropriate pedagogies that consider the children’s cognitive capacity and age [10]. Adapting content to children’s cognitive abilities and digital literacy is essential, as is aligning the course duration with expected age-appropriate attention spans [27,41,42,43]. Incorporating and delivering evidence-based and age-appropriate educational material may be challenging and often exhausting for teachers [34,35], and therefore, experts are tasked with designing and often delivering this material. The Nutritional Adventures program adopts story telling for younger students (grades 1–3) and interactive training for older students (grades 4–6), matching their cognitive capabilities and preferences. Such interactive theatrical and practical workshops, incorporating activities like stories, role-playing, and games, have effectively promoted behavioral change and dietary improvement in children [14,21,44].

The ideal duration for educational interventions remains a topic of debate. Longer durations have been associated with positive outcomes [19], with research suggesting a minimum of 40–50 classroom hours [45], six months [46], or even 12 months for long-term changes in children’s dietary habits and attitudes [19]. Nevertheless, several studies, including ours, have demonstrated that even brief interventions that can easily be incorporated into school curriculums can impact nutritional knowledge [19,21] and dietary behaviors [13,20,21]. Identifying the ideal duration should involve comprehensive planning considering the desired effect, cost-effectiveness, volume of material, and likelihood of achieving beneficial outcomes [47]. 

Maintaining children’s attention and motivation by providing engaging content is key to the program’s success. Online education should employ various multimedia resources, such as interactive presentations with videos, quizzes, and simulations [48]. Educational materials should be dynamic and visually appealing, including animations, videos, comics, and printed materials like posters or flyers, with a focus on gain-framed messages [49,50,51,52]. Another important aspect of online education is the health professional-provided feedback, offering flexible support and addressing questions or gaps following activities [26,53]. With these in mind, we developed an online educational program that combines materials provided by experts, including infographics, quizzes, and interactive activities, with creatively designed printed material.

Furthermore, educational programs prioritize fostering interactivity among students, imaginative play, and collaborative spirit [54], cultivating a sense of community and active engagement. Encouraging teamwork is paramount in this regard, allowing students to collaborate, share perspectives, and gain insights collectively [27]. Meanwhile, educators and experts can be tasked with moderating the course, ensuring that all students are actively participating and understand the material provided. However, to ensure a discrimination-free online education with all students interacting with each other, online education should be accessible to all students and all schools [27], which translates to free-of-charge access to high-quality and tailored online educational material. Keeping these principles in mind, the Nutritional Adventures program delivers an interactive educational course with engaging activities to all participating students, aided by the widespread availability of remote technology in schools.

Apart from the experts, the combined involvement in education of both schools and families should be actively involved in child education [10]. On the one hand, teachers are well-equipped with skills that can lead to student engagement and deliver the material in an age-appropriate way. Furthermore, as most social interactions and physical activities are conducted during school hours, learning can be enhanced through collaboration and socializing [24]. This was evident in our study, with teachers who oversaw the educational material during school hours presenting a higher improvement in HRQoL, encompassing aspects of school and social health. Teacher involvement is also expected to have longer long-lasting effect, as although parental involvement in nutritional education can be effective up till the age of 12, school-based intervention, often delivered or supervised by teachers, can be effective through childhood and adolescence [55]. This notion was corroborated by the conclusion that as children get older and expand their social circle, the family’s influence becomes less significant [56].

Nevertheless, the role of parental involvement in education should not be undermined. Many interventions call for the involvement of parents in the educational process [13,21]. Parental involvement is often achieved with homework assignments [12,19,57], active participation in educational activities [14,21], training sessions curated specifically for the parents [21], newsletters [58], and many more activities. Considering the determining influence of families in nutritional exposures and behaviors [59,60], the WHO guidelines for Health-Promoting Schools stresses the importance of incorporating a family component into healthy eating efforts and reinforcing nutritional knowledge in all aspects of the children’s life [10,61]. The general consensus is that school and family reinforcement should be combined to achieve the best results [10]. 

Interventions aimed at enhancing the dietary habits of children should unequivocally eschew discriminatory practices. It is imperative, in particular, to formulate interventions that exhibit no bias towards children with overweight or obesity, as the stigmatization of obesity can impede the positive outcomes of endeavors directed at the treatment and prevention of obesity [62,63]. Children with overweight or obesity often demonstrate poorer dietary habits compared to their normal-weight counterparts [64]. Nevertheless, children with overweight and obesity may become more motivated to change their habits, especially in monitoring their intake, reducing overall food consumption, limiting the intake of sweets and high-fat products, and engaging in more physical activity [65]. Furthermore, a desire for improved health, enhanced self-esteem, and positive body image, coupled with the avoidance of bullying, serves as key motivations for weight loss in children [66]. Specifically, alleviating the stigma associated with obesity, a significant psychological stressor, holds promise for improving mental health [67]. Offering guidance on enhancing dietary habits and promoting physical activity can be pivotal in fostering healthier behaviors for children with overweight and obesity, leading to improvements in both mental and physical well-being, as evidenced by the findings in our study.

Online education is a dynamic and evolving domain, characterized by various emerging trends. One such trend is the incorporation of gamification and “serious games” into nutritional education. In gamified systems, various game-related elements that are not limited to scoring systems, leaderboards, badges, and time pressure can be embedded into the activities to ensure engagement and enhance motivation [68,69]. On the other hand, digital games with a more “serious” intent are designed with the purpose of offering learning skills, training, and acquiring new and enhanced behaviors [70]. Both approaches have been considered effective at improving children’s dietary habits and nutrition literacy [71], with the potential of harnessing technological innovations like virtual reality, which has shown promise in influencing children’s eating behavior [72]. 

Since many of these approaches are relatively recent, it is imperative to investigate their long-term effects and compare them with traditional educational methods. Assessing enduring changes in dietary habits is a key outcome, but researchers should also consider improvements in other areas that are often affected, such as HRQoL, mental health, and behavioral changes within families [13,14,15,16,17,73]. Researching the sustainability of these changes and communicating this knowledge to health professionals and game developers are crucial steps [71]. 

Despite the demonstrated efficacy of our program, the limitations of our study should not be ignored. Firstly, the program had a relatively short duration, encompassing two school hours of interactive educational activities and a limited amount of time during which students engaged with supplementary educational materials. Nevertheless, notwithstanding its brief duration, the program exhibited a notable efficacy in enhancing children’s dietary habits and HRQoL, promoting physical activities, and restricting screen time. The statistical analysis was conducted with data from the intervention schools and no control group to compare. In order to verify that the observed changes result from our intervention and to what extent, controlled studies should be organized. Nevertheless, as the baseline and follow-up questionnaires were completed within 3–5 weeks, no major changes in dietary habits or HRQoL should be expected for those not receiving the intervention. Despite the reported abstention, our final sample size (*n* = 1487) was adequately powered (statistical power = 80%) to assess the detected differences at a statistical significance level of less than 0.05. In addition to this, no statistically significant differences were observed between responders and non-responders in terms of the school grade. Additionally, there are various conditions that could have affected students’ quality of life, acting as potential confounders in our research hypothesis; nevertheless, the follow-up time is short enough to let us assume that the observed changes are attributed principally to our intervention. Lastly, although the COVID-19 pandemic has made online resources more accessible, the appropriate equipment to support our virtual program might not be available to all schools in Greece. Taking the above into consideration, a short-term virtual intervention can be effective in disseminating nutritional information to school-aged children and affect their dietary habits; however, in order to corroborate the results, controlled studies and long-term follow-up are indicated.

## 5. Conclusions

The present study demonstrates that the “Food Adventures” (‘Diatrofoperipeties’) program successfully improved dietary and lifestyle habits and HRQoL in primary school children (6–12 years old). Designing a school-based virtual curriculum enabled the widespread dissemination of nutrition education across Greece, overcoming geographic and financial limitations, while the one-month duration allowed for easy integration into the school curriculum. Additionally, the child-centered approach and inclusion of either parent or educator supervision proved efficient. However, further studies with control groups and longer follow-up times are necessary to confirm the effectiveness of such programs.

## Figures and Tables

**Table 1:** Baseline characteristics of students in the total sample and according to students’ grades.

Baseline Characteristics of Students	Total Sample	1st–3rd Grade	4th–6th Grade	*p*
N	1487	804	674	
Age, Mean (SD)	9 (2)	7 (1)	10 (1)	<0.001
Boys, %	45.1	46.1	44.5	0.544
Non-immigrant father, %	84.4	85.2	83.5	0.378
Non-immigrant mother, %	82.8	83.3	82.3	0.615
Paternal educational level, %				
High (>12 years)	50.8	50.1	51.6	
Moderate (9–12 years)	33.5	34.4	32.5	0.773
Low (<9 years)	15.7	15.5	15.9	
Maternal educational level, %				
High (>12 years)	69.6	71.2	67.6	
Moderate (9–12 years)	23.6	22.3	25.1	0.427
Low (<9 years)	6.8	6.5	7.3	
Parental income status, %				
Both parents with income	69.2	68.6	69.9	
One parent with income	29.7	30.5	28.7	0.534
Both parents without income	1.1	0.9	1.4	
At least two siblings, %	25.3	22.7	28.0	0.020

Data are presented as mean (standard deviation) for normally distributed continuous variables (age) and % of the corresponding sample for categorical variables. For the normally distributed variables (age), p’s were obtained using two-sample *t*-test. For the categorical variables, chi-squared test was performed. Nine students did not report their grade (*n* = 9 missing values). Abbreviations: Standard Deviation (SD); *p*-value (*p*).

**Table 2:** Level of adherence to the Mediterranean diet, dietary habits, physical activity, and screen time at baseline and follow-up according to students’ grade.

Outcomes	Total Sample (N = 1487)			1st–3rd Grade (N = 804)			4th–6th Grade (N = 674)		
	Baseline	Follow-Up	*p*	Baseline	Follow-Up	*p*	Baseline	Follow-Up	*p*
KIDMED score	5.5 (2.3)	5.7 (2.4)	<0.001	5.5 (2.3)	5.8 (2.3)	<0.001	5.4 (2.4)	5.6 (2.5)	0.003
Adherence to the Mediterranean diet, %									
Poor	20.4	18.3	0.021	18.2	16.9	0.141	22.8	19.8	0.152
Average	60.9	60.3		63.1	61.6		58.4	59.1	
Good	18.7	21.3		18.7	21.6		18.8	21.1	
Consumption of, %									
Fruit 1/day	84.0	86.1	0.041	85.0	87.2	0.112	83.0	84.9	0.241
Fruit > 1/day	39.7	42.4	0.042	39.0	42.2	0.074	40.5	42.9	0.244
Vegetables 1/day	62.3	65.9	0.009	64.1	68.3	0.013	60.1	63.2	0.182
Vegetables > 1/day	24.2	28.4	0.004	25.3	28.7	0.084	22.6	28.2	0.010
Fish ≥ 2–3/week	30.4	32.1	0.221	30.5	31.2	0.719	30.4	33.4	0.144
Fast food > 1/week	11.3	13.2	0.075	11.0	13.0	0.163	11.6	13.6	0.208
Pulses > 1/week	69.5	67.8	0.185	71.8	70.7	0.482	66.6	64.0	0.190
Commercially baked goods or pastries for breakfast	57.2	59.9	0.106	58.5	61.1	0.237	55.4	58.4	0.233
Two yoghurts and/or 40 g cheese daily	47.8	51.1	0.041	49.9	51.4	0.481	45.3	50.9	0.023
Sweets and candy several times a day	28.2	25.7	0.054	28.3	25.8	0.151	28.0	25.4	0.201
Daily breakfast	77.3	77.9	0.604	79.6	80.9	0.346	74.8	74.1	0.701
Physical activity (hours/week)	3.1 (2.1)	3.4 (2.2)	<0.001	3.0 (1.9)	3.3 (2.1)	<0.001	3.2 (2.2)	3.6 (2.4)	<0.001
Screen time (hours/week)	6.1 (2.9)	5.6 (2.9)	<0.001	5.9 (2.9)	5.3 (2.8)	<0.001	6.5 (2.9)	5.9 (2.9)	<0.001

Quantitative variables are presented as mean (sd). Categorical outcomes are presented as proportion to the total outcome group population. Dietary habits emerged from KIDMED questions. *p*-values were obtained using paired χ^2^ for categorical variables and paired *t*-test for quantitative variables. Abbreviations: *p*-value (*p*); Standard deviation (sd); Grams (g).

**Table 3:** Students’ health-related quality of life at baseline and follow-up in the total sample and according to students’ grade.

Outcomes	Total Sample (N = 1487)			1st–3rd Grade (N = 804)			4th–6th Grade (N = 674)			*p* (Group Difference) *
	Baseline, M (IQR)	Difference, Mean (SD)	*p*	Baseline, M (IQR)	Difference, Mean (SD)	*p*	Baseline, M (IQR)	Difference, Mean (SD)	*p*	
HRQoL	92 (84–97)	1.35 (7.82)	<0.001	92 (85–97)	1.60 (7.20)	<0.001	92 (84–97)	0.94 (8.38)	0.004	0.106
Physical functioning	96 (87–100)	0.99 (10.2)	<0.001	94 (88–100)	1.56 (8.92)	<0.001	97 (88–100)	0.18 (11.3)	0.686	0.009
Emotional functioning	85 (75–95)	2.15 (12.7)	<0.001	85 (75–95)	2.12 (12.0)	<0.001	85 (75–95)	1.92 (13.5)	<0.001	0.656
Social functioning	95 (85–100)	0.84 (10.9)	0.003	95 (90–95)	0.94 (10.7)	0.014	100 (85–100)	0.69 (11.0)	0.053	0.671
School functioning	95 (85–100)	1.58 (10.3)	<0.001	95 (85–100)	1.62 (10.00)	<0.001	95 (80–100)	1.46 (10.7)	<0.001	0.770

All baseline outcomes are presented as median (IQR) due to non-normal distribution. All differences were calculated as follow-up score—baseline score and are presented as mean (SD) due to normal distribution. *p*-values were obtained using one-sample *t*-test for all baseline and follow-up comparisons for quantitative variables (null hypothesis: mean difference = 0). * Two-sample *t*-test was used to compare each difference between 1st–3rd grade students and 4th–6th grade students. Nine students did not report their grade (*n* = 9 missing values). Abbreviations: Median (M); Standard Deviation (SD); Interquartile range (IQR); *p*-value (*p*).

**Table 4:** Level of adherence to the Mediterranean diet, dietary habits, physical activity, and screen time at baseline and follow-up according to intervention group (parent and educator).

Outcomes	Parent Group (N = 714)			Educator Group (N = 773)		
	Baseline	Follow-Up	*p*	Baseline	Follow-Up	*p*
KIDMED score	5.5 (2.4)	5.8 (2.4)	<0.001	5.4 (2.3)	5.6 (2.4)	0.002
Adherence to the Mediterranean diet, %						
Poor	19.6	18.3	0.251	20.6	17.5	0.109
Average	61.3	59.1		60.5	62.1	
Good	19.1	22.6		18.9	20.3	
Consumption of, %						
Fruit 1/day	83.3	85.8	0.074	84.5	86.4	0.245
Fruit > 1/day	39.7	43.5	0.048	39.7	41.3	0.371
Vegetables 1/day	60.2	65.1	0.012	64.4	66.7	0.293
Vegetables > 1/day	24.1	29.3	0.015	24.2	27.6	0.092
Fish ≥ 2–3/week	30.8	32.4	0.454	30.0	31.8	0.322
Fast food > 1/week	10.9	11.9	0.529	11.7	14.5	0.059
Pulses > 1/week	69.9	69.2	0.663	69.1	66.5	0.162
Commercially baked goods or pastries for breakfast	56.1	60.0	0.098	58.3	59.8	0.519
Two yoghurts and/or 40 g cheese daily	48.9	52.1	0.149	46.7	50.1	0.147
Sweets and candy several times a day	27.6	24.9	0.174	28.9	26.4	0.173
Daily breakfast	78.5	78.4	0.907	76.2	77.5	0.370
Physical activity (hours/week)	3.2 (2.1)	3.5 (2.3)	<0.001	3.0 (2.0)	3.3 (2.2)	<0.001
Screen time (hours/week)	6.3 (3.0)	5.6 (2.9)	<0.001	6.0 (2.9)	5.6 (2.9)	<0.001

Quantitative variables are presented as mean (sd). Categorical outcomes are presented as proportion of the total outcome group population. Dietary habits emerged from KIDMED questions. *p*-values were obtained using paired χ^2^ for categorical variables and paired *t*-test for quantitative variables. Abbreviations: *p*-value (*p*); Standard deviation (sd); Grams (g).

**Table 5:** Students’ health-related quality of life at baseline and follow-up according to the intervention group (parent and educator).

Outcomes	Parent Group (N = 714)			Educator Group (N = 773)			*p* (Group Difference) *
	Baseline, M (IQR)	Difference, Mean (SD)	*p*	Baseline, M (IQR)	Difference, Mean (SD)	*p*	
HRQoL	92 (85–97)	0.98 (7.66)	<0.001	91 (83–96)	1.69 (7.95)	<0.001	0.079
Physical functioning	97 (88–100)	0.57 (9.19)	0.098	94 (88–100)	1.37 (11.1)	<0.001	0.132
Emotional functioning	85 (75–95)	1.75 (12.6)	<0.001	85 (75–95)	2.51 (12.9)	<0.001	0.248
Social functioning	100 (88–100)	0.45 (11.0)	0.280	95 (85–100)	1.22 (10.8)	0.002	0.174
School functioning	95 (85–100)	1.40 (10.1)	<0.001	94 (81–100)	1.76 (10.5)	<0.001	0.502

All baseline outcomes are presented as median (IQR) due to non-normal distribution. All differences were calculated as follow-up score—baseline score and are presented as mean (SD) due to normal distribution. *p*-values were obtained using one-sample *t*-test for all baseline and follow-up comparisons for quantitative variables (null hypothesis: mean difference = 0). * Two-sample *t*-test was used to compare each difference between 1st–3rd grade students and 4th–6th grade students. Nine students did not report their grade (*n* = 9 missing values). Abbreviations: Median (M); Standard deviation (SD); Interquartile range (IQR); *p*-value (*p*).

**Table 6:** Level of adherence to the Mediterranean diet, dietary habits, physical activity, and screen time at baseline and follow-up according to their BMI at baseline.

Outcomes	Healthy BMI at Baseline (N = 596)			Unhealthy BMI at Baseline (N = 387)		
	Baseline	Follow-Up	*p*	Baseline	Follow-Up	*p*
KIDMED score	5.7 (2.3)	5.8 (2.3)	0.539	5.1 (2.5)	5.4 (2.6)	0.005
Adherence to the Mediterranean diet, %						
Poor	15.7	14.4	0.751	26.4	23.8	0.242
Average	63.1	63.8		56.1	57.4	
Good	32.2	21.7		17.5	18.8	
Consumption of, %						
Fruit 1/day	86.5	88.4	0.258	77.9	82.2	0.070
Fruit > 1/day	41.8	45.0	0.113	35.9	39.2	0.189
Vegetables 1/day	63.7	64.8	0.574	61.9	64.1	0.405
Vegetables > 1/day	26.3	27.6	0.532	23.1	31.0	0.006
Fish ≥ 2–3/week	29.9	28.5	0.508	29.9	35.9	0.015
Fast food > 1/week	11.0	12.8	0.276	12.1	15.3	0.117
Pulses > 1/week	72.8	69.4	0.092	64.1	62.3	0.492
Commercially baked goods or pastries for breakfast	54.6	58.7	0.092	58.1	59.8	0.600
Two yoghurts and/or 40 g cheese daily	50.7	50.7	>0.999	49.5	51.6	0.513
Sweets and candy several times a day	26.3	24.2	0.335	28.1	26.0	0.431
Daily breakfast	80.6	81.1	0.777	73.7	71.5	0.355
Physical activity (hours/week)	3.3 (2.1)	3.6 (2.3)	<0.001	3.0 (2.0)	3.3 (2.2)	0.002
Screen time (hours/week)	6.0 (2.9)	5.6 (2.8)	<0.001	6.4 (2.9)	5.8 (2.9)	<0.001

Quantitative variables are presented as mean (sd). Categorical outcomes are presented as proportion of the total outcome group population. Dietary habits emerged from KIDMED questions. *p*-values were obtained using paired χ^2^ for categorical variables and paired *t*-test for quantitative variables. Abbreviations: *p*-value (*p*); Standard deviation (sd); Grams (g).

**Table 7:** Students’ health-related quality of life at baseline and follow-up according to their BMI at baseline.

Outcomes	Healthy BMI at Baseline (N = 596)			Unhealthy BMI at Baseline (N = 387)			*p* (Group Difference) *
	Baseline, M (IQR)	Difference, Mean (SD)	*p*	Baseline, M (IQR)	Difference, Mean (SD)	*p*	
HRQoL	92 (86–97)	0.71 (6.47)	0.008	91 (82–96)	1.65 (9.69)	0.001	0.070
Physical functioning	97 (91–100)	−0.03 (8.35)	0.932	94 (84–100)	1.38 (12.47)	0.030	0.035
Emotional functioning	85 (75–95)	1.53 (11.86)	0.002	85 (75–95)	3.19 (13.3)	<0.001	0.044
Social functioning	100 (90–100)	0.48 (9.26)	0.204	95 (85–100)	1.02 (13.1)	0.127	0.452
School functioning	95 (85–100)	1.09 (9.19)	0.004	90 (85–100)	1.43 (11.74)	0.017	0.615

All baseline outcomes are presented as median (IQR) due to non-normal distribution. All differences were calculated as follow-up score—baseline score and are presented as mean (SD) due to normal distribution. *p*-values were obtained using one-sample *t*-test for all baseline and follow-up comparisons for quantitative variables (null hypothesis: mean difference = 0). * Two-sample *t*-test was used to compare each difference between students with healthy and unhealthy weights. Abbreviations: Median (M); Standard deviation (SD); Interquartile range (IQR); *p*-value (*p*); Body Mass Index (BMI).

## Data Availability

All data and materials are available upon reasonable request to the corresponding author.

## Appendix A

**Table A1:** Baseline characteristics of students in the total sample and according to intervention group (parent and teacher).

Baseline Characteristics of Students	Parent Group	Educator Group	*p*
N	714	773	
Age, Mean (SD)	9 (2)	9 (2)	0.831
Boys, %	45.5	44.8	0.766
Non-immigrant father, %	87.3	81.8	0.004
Non-immigrant mother, %	84.7	81.0	0.056
Paternal educational level, %			
High	51.0	50.7	
Moderate	33.5	33.5	0.982
Low	15.5	15.9	
Maternal educational level, %			
High	70.4	68.7	
Moderate	21.6	25.6	0.131
Low	8.0	5.7	
Parental income status, %			
Both parents with income	68.5	69.8	
One parent with income	30.5	29.0	0.773
Both parents without income	1.0	1.2	
At least 2 siblings, %	25.5	25.1	0.873

Data are presented as mean (standard deviation) for normally distributed continuous variables (age) and % of the corresponding sample for categorical variables. For the normally distributed variables (age), p’s were obtained using two-sample *t*-test. For the categorical variables, chi-squared test was performed. Abbreviations: Standard Deviation (SD); *p*-value (*p*).

## Appendix B

**Table A2:** Demographic characteristics of students according to intervention group (parent and teacher), by 1st–3rd grade and 4th–6th grade students separately.

Baseline Characteristics of Students	1st–3rd Grade			4th–6th Grade		
	Parent Group	Educator Group	*p*	Parent Group	Educator Group	*p*
N	383	421	-	327	347	-
Age, Mean (SD)	7.4 (0.9)	7.2 (1.0)	0.030	9.9 (0.9)	10.1 (0.9)	<0.001
Boys, %	41.5	50.2	0.014	50.9	38.4	<0.001
Non-immigrant father, %	87.7	82.9	0.054	86.9	80.4	0.024
Non-immigrant mother, %	84.9	81.9	0.269	85.0	79.8	0.077
Paternal educational level, %						
High	52.3	48.2		49.7	53.4	
Moderate	32.5	36.0	0.540	34.4	30.7	0.602
Low	15.2	15.7		16.0	15.9	
Maternal educational level, %						
High	75.5	67.2		64.8	70.4	
Moderate	18.1	26.2	0.058	25.3	24.9	0.076
Low	6.4	6.6		9.9	4.7	
Parental income status, %						
Both parents with income	68.3	68.9		69.2	70.6	
One parent with income	30.5	30.5	0.686	30.1	27.3	0.319
Both parents without income	1.2	0.6		0.7	2.1	
At least 2 siblings, %	23.8	21.6	0.465	27.1	28.8	0.630

Data are presented as mean (standard deviation) for normally distributed continuous variables (age) and % of the corresponding sample for categorical variables. For the normally distributed variables (age), p’s were obtained using two-sample *t*-test. For the categorical variables, chi-squared test was performed. Abbreviations: Standard Deviation (SD); *p*-value (*p*).

## Appendix C

**Table A3:** Demographic characteristics of students according to their grade, by intervention group (parent and teacher).

Baseline Characteristics of Students	Parent Group			Educator Group		
	1st–3rd Grade	4th–6th Grade	*p*	1st–3rd Grade	4th–6th Grade	*p*
N	383	327	-	421	347	-
Age, Mean (SD)	7.4 (0.9)	9.9 (0.9)	<0.001	7.2 (1.0)	10.1 (0.9)	<0.001
Boys, %	41.5	50.9	0.014	50.2	38.4	<0.001
Non-immigrant father, %	87.7	86.9	0.736	82.9	80.4	0.399
Non-immigrant mother, %	84.9	85.0	>0.999	81.9	79.8	0.461
Paternal educational level, %						
High	52.3	49.7		48.2	53.4	
Moderate	32.5	34.4	0.804	36.0	30.7	0.318
Low	15.2	16.0		15.7	15.9	
Maternal educational level, %						
High	75.5	64.8		67.2	70.4	
Moderate	18.1	25.3	0.024	26.2	24.9	0.570
Low	6.4	9.9		6.6	4.7	
Parental income status, %						
Both parents with income	68.3	69.2		68.9	70.6	
One parent with income	30.5	30.1	0.838	30.5	27.3	0.171
Both parents without income	1.2	0.7		0.6	2.1	
At least 2 siblings, %	23.8	27.1	0.338	21.6	28.8	0.028

Data are presented as mean (standard deviation) for normally distributed continuous variables (age) and % of the corresponding sample for categorical variables. For the normally distributed variables (age), p’s were obtained using two-sample *t*-test. For the categorical variables, chi-squared test was performed. Abbreviations: Standard Deviation (SD); *p*-value (*p*).

## Appendix D

**Table A4:** Changes in the adherence to the Mediterranean diet, HRQoL, physical activity, and screen time between baseline and follow-up (difference), according to students’ grade, by intervention group (parent and teacher) separately.

Outcomes	Parent Group			Educator Group		
	1st–3rd Grade	4th–6th Grade	*p*	1st–3rd Grade	4th–6th Grade	*p*
N	383	327	-	421	347	-
Mean (SD) difference in						
KIDMED score	0.21 (1.69)	0.33 (1.80)	0.423	0.26 (1.59)	0.19 (1.95)	0.678
HRQoL	1.07 (6.24)	0.80 (9.07)	0.653	2.08 (7.95)	1.07 (7.66)	0.077
Physical functioning	0.81 (7.70)	0.22 (10.7)	0.396	2.25 (9.85)	0.14 (11.9)	0.008
Emotional functioning	1.81 (11.1)	1.54 (14.1)	0.778	2.58 (12.9)	2.27 (12.9)	0.741
Social functioning	0.53 (10.1)	0.38 (12.0)	0.860	1.31 (11.2)	0.99 (10.1)	0.685
School functioning	1.20 (8.58)	1.52 (11.6)	0.688	1.99 (11.1)	1.40 (9.68)	0.442
Improved their adherence to the Mediterranean diet, %	19.1	18.5	0.852	15.7	19.5	0.257
Increased their physical activity, %	16.6	11.3	0.054	16.0	20.1	0.158
Reduced their screen time, %	6.4	3.6	0.097	4.6	5.7	0.510

All differences were calculated as follow-up score—baseline score and are presented as mean (SD) due to normal distribution. p’s were obtained using two-sample *t*-test for qualitative variables and χ^2^ for categorical variables. Improvement in adherence to the Mediterranean diet was defined as baseline poor adherence to moderate/high at follow-up or moderate at baseline to high at follow-up. Abbreviations: Median (M); Standard deviation (SD); Interquartile range (IQR); *p*-value (*p*).

## Appendix E

**Table A5:** Changes in the adherence to the Mediterranean diet, dietary habits, HRQoL, physical activity, and screen time between baseline and follow-up (difference), according to intervention group (parent and educator), by 1st–3rd grade and 4th–6th grade students separately.

Outcomes	1st–3rd Grade			4th–6th Grade		
	Parent Group	Educator Group	*p*	Parent Group	Educator Group	*p*
N	383	421	-	327	347	-
Mean (SD) difference in:						
KIDMED score	0.21 (1.69)	0.26 (1.59)	0.744	0.33 (1.80)	0.19 (1.95)	0.408
HRQoL	1.07 (6.24)	2.08 (7.95)	0.047	0.80 (9.07)	1.07 (7.66)	0.677
Physical functioning	0.81 (7.70)	2.25 (9.85)	0.022	0.22 (10.7)	0.14 (11.9)	0.926
Emotional functioning	1.81 (11.1)	2.58 (12.9)	0.368	1.54 (14.1)	2.27 (12.9)	0.487
Social functioning	0.53 (10.1)	1.31 (11.2)	0.305	0.38 (12.0)	0.99 (10.1)	0.479
School functioning	1.20 (8.58)	1.99 (11.1)	0.267	1.52 (11.6)	1.40 (9.68)	0.892
Improved their adherence to the Mediterranean diet, %	19.1	15.7	0.280	18.5	19.5	0.778
Increased their physical activity, %	16.6	16.0	0.835	11.3	20.1	0.003
Reduced their screen time, %	6.4	4.6	0.282	3.6	5.7	0.204
Improved daily fruit intake, %	59 (15.4)	68 (16.2)	0.772	53 (16.2)	52 (115)	0.662
Improved daily vegetable intake, %	78 (20.4)	83 (19.7)	0.818	72 (22.0)	66 (19.0)	0.335

All differences were calculated as follow-up score—baseline score and are presented as mean (SD) due to normal distribution. p’s were obtained using two-sample *t*-test for qualitative variables and χ^2^ for categorical variables. Improvement in adherence to the Mediterranean diet was defined as baseline poor adherence to moderate/high at follow-up or moderate at baseline to high at follow-up. Abbreviations: Median (M); Standard deviation (SD); Interquartile range (IQR); *p*-value (*p*).
